# Polymer–Graphene Composites for Electrochemical Sensing: A Comprehensive Review of Functionalization Pathways and Sustainable Design Strategies

**DOI:** 10.3390/polym18091120

**Published:** 2026-05-01

**Authors:** Domingo César Carrascal-Hernández, Andrea Ramos-Hernández, Nataly J. Galán-Freyle, Daniel Insuasty, Maximiliano Méndez-López

**Affiliations:** 1 Grupo de Investigación en Sociedad, Educación y Desarrollo Humano GISEDH, Facultad de Ciencias, Educación, Artes y Humanidades, Institución Universitaria de Barranquilla (IUB), Barranquilla 080002, Colombia; 2 Grupo Química Supramolecular Aplicada, Semillero Electroquímica Aplicada, Facultad de Ciencias Básicas, Programa de Química, Universidad del Atlántico, Barranquilla 081007, Colombia; andrearamos@mail.uniatlantico.edu.co; 3 Facultad de Ciencias Básicas y Biomédicas, Life Science Research Center, Universidad Simón Bolívar, Barranquilla 080002, Colombia; 4 Departamento de Química y Biología, Facultad de Ciencias Básicas, Universidad del Norte, Barranquilla 080020, Colombia; insuastyd@uninorte.edu.co (D.I.); maximilianom@uninorte.edu.co (M.M.-L.)

**Keywords:** electrochemical sensors, graphene, hybrid nanomaterials, graphene oxide, reduced graphene oxide

## Abstract

Environmental pollution constitutes an increasingly complex global challenge, largely driven by industrial expansion and the consequent release of toxic species such as Cd^2+^, Pb^2+^, Cu^2+^, Hg^2+^, Fe^3+^, As^3+^, and Rh^3+^ into natural ecosystems. These contaminants pose significant risks to environmental integrity and public health, motivating the development of analytical technologies capable of sensitive, selective, and reliable detection. In this context, graphene-based electrochemical sensors have emerged as versatile platforms for monitoring a broad range of analytes, particularly in environmental applications involving heavy-metal detection. The intrinsic physicochemical properties of graphene derivatives have enabled low detection limits, rapid response times, and tunable selectivity. Despite analytical advances, critical challenges persist regarding operational stability in complex matrices, inter-batch reproducibility, and robustness to interfering species, which continue to hinder large-scale deployment and real-world applicability. However, challenges remain regarding stability and performance in complex arrays, reproducibility, and resistance to interference, necessitating innovative strategies for functionalization and molecular recognition. This review article establishes a comparative framework based on functionalization strategies (covalent, non-covalent, and hybrid), the chemical nature of graphene (GO, rGO, and doping), and various types of polymers (conductors and insulators), using statistical metrics such as the limit of detection (LOD), linear range, working potential, stability, and interferences, employing a bibliometric analysis using the PRISMA 2020 methodology. This comparative framework enables analysis and explanation of performance trends, and the generation of design and functionalization recommendations for versatile applications, including criteria for reproducibility and sustainability.

## 1. Introduction

Two-dimensional (2D) materials such as graphene are composed of a single layer of carbon atoms (0.334 nm thick) arranged in a hexagonal lattice. They exhibit high electron mobility (>200,000 cm^2^/V·s), a high Young’s modulus (~1 TPa), high thermal conductivity (3000–5000 W/m·K), high specific surface area (~2600 m^2^/g), and high optical transparency (97.4%), among other properties, which are of interest in various research fields [1,2]. On the other hand, polymers (macromolecular systems formed by long chains of repeating units called monomers) are materials of natural origin (cellulose, starch, rubber) or semi-synthetic or synthetic origin (PVC, nylon, polyethylene, polystyrene). They represent an extremely diverse structural range, enabling the design of smart polymers (sensitive to temperature, pH, or light) and recyclable/sustainable polymers and materials that self-repair [3,4]. These two materials (graphene and certain polymers) can form hybrid materials with improved electronic properties and performance that exceed those of their individual components, resulting in a broad spectrum of materials with unique properties [5].

However, the industrial-scale production of high-quality graphene is complex and has remained a challenge in recent years. For example, methods such as exfoliation, chemical vapor deposition (CVD), and reduction of graphene oxide (rGO) exhibit low reproducibility or high costs, limiting their industrial use [2]. Furthermore, structural defects such as vacancies, zigzag/armchair edge irregularities, and contamination affect electron transport, reducing mobility and conductivity [4]. Similarly, most polymers are insulators; even conductive polymers depend on doping or highly ordered structures, which are not always stable or scalable [6]. In addition, they exhibit aging, degradation mediated by temperature, UV radiation, humidity, oxidation, etc., which affect their electronic properties [7]. In recent years, various environmental challenges have driven significant research (particularly in the field of high-performance electrochemical device design) aimed at developing sensors capable of meeting increasingly stringent requirements. This progress is closely linked to the growing need to detect biomarkers, metabolites, and environmental pollutants with high sensitivity and minimal latency, a challenge on a global scale [8]. Added to this is the pressure exerted by the fields of microelectronics and nanotechnology, where miniaturization and portability are no longer desirable attributes, but essential requirements for the development of intelligent and low-energy platforms [9,10]. In this context, hybrid systems based on functional polymers and graphene have become established as a particularly promising strategy [11,12,13]. The contribution of graphene opens the possibility of manufacturing sensors with active surfaces that exhibit high electron mobility, high surface area, and exceptional interfacial connectivity—essential features for improving charge-transfer processes in advanced electrochemical platforms [14].

The functionalization of graphene–polymer materials and/or composites can be approached through three main strategies, each with specific implications for performance, stability, and adaptability.

(1) Covalent functionalization: This approach involves the chemical modification of graphene or its derivatives (GO, rGO) through reactions such as amidation, esterification, or acylation, allowing the incorporation of functional groups such as –COOH, –NH_2_, epoxy groups, –SO_3_^−^, among others [15]. These groups serve as anchoring sites for conductive polymers (polyaniline, polypyrrole) or biopolymers (chitosan, alginate), creating a robust and stable interface. Although this strategy may disrupt the π-conjugation of graphene and reduce its conductivity, it offers advantages in terms of durability, mechanical strength, and compatibility with biological matrices [16].

(2) Non-covalent functionalization, based on supramolecular interactions (π–π stacking, hydrogen bonding, Van der Waals forces, electrostatic interactions), is particularly appealing because it preserves the structural and electronic integrity of materials such as graphene, maintaining its high charge mobility [17]. Moreover, it enables the self-assembly of polymers on the graphene surface, facilitating the reversible incorporation of biomolecules such as enzymes, antibodies, or aptamers [18]. This reversibility is especially advantageous for reusable or regenerable sensors, where system integrity must be maintained after multiple operational cycles [19].

(3) Hybrid functionalization and other emerging approaches combine the best of both worlds: the structural stability of covalent functionalization and the versatility of non-covalent interactions [20,21]. In this regard, advanced methodologies have emerged, such as directed electropolymerization [22], click chemistry [23], self-assembly assisted by electric or magnetic fields [24], and solid-phase functionalization [25]. These techniques enable the construction of highly organized interfaces with specific active sites for biological, environmental, or industrial analytes, even under conditions of chemical interference or thermal variability.

For example, Hamdi et al. reported a comparative study involving natural polymers grafted with graphene nanoplatelets and dispersed nanofluids to enhance oil recovery in environmental spills, achieving a 17% improvement in recovery rate [26]. Similarly, Hegazy et al. investigated starch-based copolymers grafted with polyacrylamide, Fe_3_O_4_, and graphene oxide, forming nanocomposites that exhibited a high capacity for nickel (Ni) removal from industrial effluents at low cost, with an adsorption capacity of 290 mg/g [27]. These structures not only optimize charge transfer and chemical compatibility but also allow the incorporation of specific functionalities, such as molecular recognition [28], responsiveness to external stimuli, and structural regeneration capabilities, including applications in regenerative medicine [29].

The choice of functionalization strategy should not be addressed in isolation. It depends on the type of polymer, the target analyte, the operational environment, and the required detection conditions. Despite various technical advances, conceptual fragmentation persists in the literature, hindering the development of a unified theoretical framework for the preparation, characterization, and application of these materials in electrochemical sensors [30]. This review seeks to bridge that gap through a systematic, multidisciplinary analysis integrating perspectives from materials chemistry, sensor engineering, and the applied sciences. The goal is not only to describe what has been achieved but also to identify what remains unresolved: How can we design sensors that not only detect but also learn from their environment? How can reproducibility be ensured in complex biological matrices? To address these questions, we conducted a bibliometric analysis using the PRISMA 2020 methodology over the last decade to broaden the scope of research on graphene–polymer-based electrochemical sensors. Additionally, a search key was designed using the PICO methodology with DeCS/MeSH descriptors and Boolean operators. This key was applied to databases such as Scopus and Web of Science, limiting the results to articles, reviews, English-language publications, and the year. Duplicates and articles unrelated to chemical sensors, GO, rGO, etc., were excluded. Bibliometric mapping was performed using VOSviewer v1.6.18, which enabled analysis of co-authorship, co-occurrence, and co-citation among the retrieved articles, thereby identifying current trends in this field.

## 2. Methodology

In this review article, we provide a comprehensive analysis of the current state of polymer–graphene composite systems, functionalization pathways, and sustainable design strategies to produce high-efficiency electrochemical sensors. We focus on the preparation, properties, and multidisciplinary applications of these systems over the past decade (2015–2025), following the PRISMA 2020 methodology (Preferred Reporting Elements for Systematic Reviews and Meta-Analyses) [31]. Elsevier Scopus was the database of choice in this context, as it was used to thoroughly examine citation and abstract information from scientific journals. Given its extensive coverage of a wide range of publishers and journals, Scopus is widely regarded as a primary source of scientific information. Moreover, it hosts over 25,100 titles from more than 5000 international publishers [32], and the Web of Science (WoS) database, a comprehensive scientific repository with more than 34,000 journals selected according to quality criteria, was also used [33].

The impact factor (IF) of the journals for the articles included in the bibliometric analysis was obtained from the Journal Citation Reports (JCR) 2024 (WoS) and CiteScore 2022 from Scopus. Additionally, the open-source software VOSviewer (www.vosviewer.com; Van Eck and Waltman, 2009–2022, version 1.6.18, Leiden University, Leiden, The Netherlands) was used in this review article to create network maps of institutions, countries, keywords, and citations per article, which is appropriate for mapping all the information related to the use of materials such as graphene and various polymers used for the development of high-efficiency sensors [34]. We accessed VOSviewer on 20 November 2025.

To ensure methodological rigor and comprehensive coverage of all articles published in the last decade related to the development and applications of polymer–graphene composites and functional materials in the design of electrochemical sensors, the PICO strategy (Population/Problem, Intervention, Comparison, Outcome) was employed to structure the literature search and guarantee broader coverage. Table 1 presents the development of the search key; this procedure integrated Boolean operators, DeCS descriptors [35], and MeSH terms [36] to maximize the scope and quality of the retrieved studies. The final search strategy was applied in Scopus and WoS, two databases recognized for their breadth and scientific reliability.

**Figure 1:**
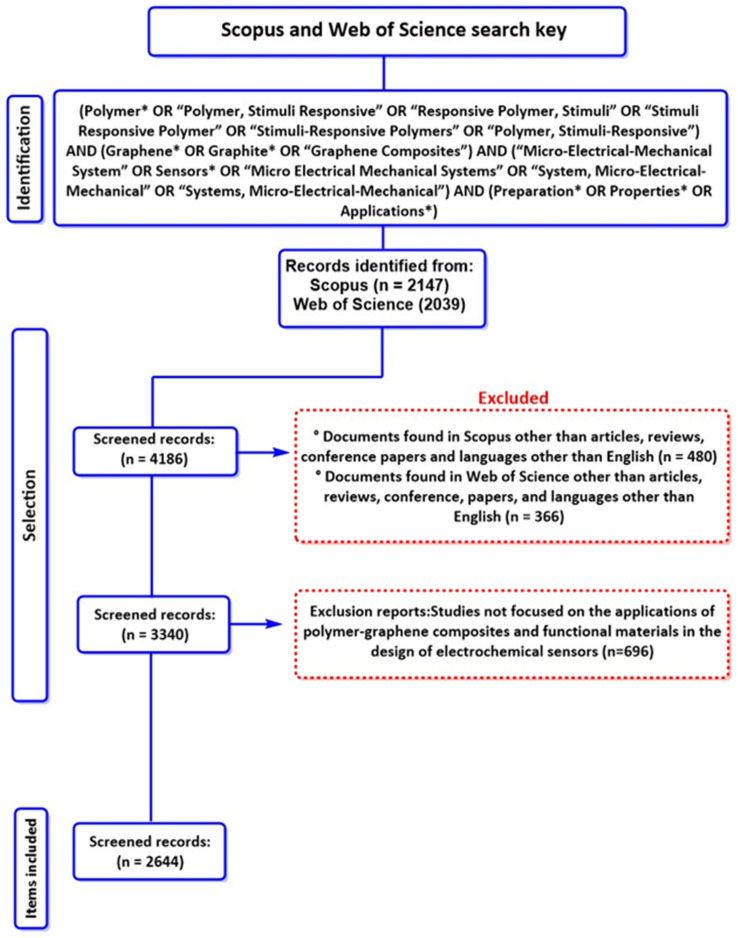
PRISMA Flow Diagram of the Methodology Used in the Systematic Review with Bibliometric Analysis. The symbol (*) indicates the inclusion of plurals in the search key terms.

Using this search key, 2147 articles were retrieved from Scopus and 2039 from WoS (accessed on 20 November 2025). The selection criteria focused on highlighting articles from the last decade, as it is essential to report and discuss the development and applications of polymer–graphene composites, as well as the various functional materials employed in electrochemical sensor design. The most recent publications in this area include novel advances and improvements that address limitations identified in previous studies. Inclusion criteria included filtering by title, abstract, and keywords; restricting the document type to “article,” “review,” or “conference paper”; and limiting the language to English, as it is the universal language of scientific publication. The exclusion criteria were meticulously designed to ensure the integrity of the study’s findings. This process entailed removing all keywords deemed unrelated to the article’s primary objective. This procedure was implemented to eliminate duplicate reports and prevent information bias. As illustrated in Figure 1, the selection of the most relevant results is based on three factors: their focus, methodological significance, and potential for industrial scalability.

## 3. Results and Discussion

### 3.1. Collaboration Between Countries on the Development of Technologies Based on Graphene and Its Derivatives

**Figure 2:**
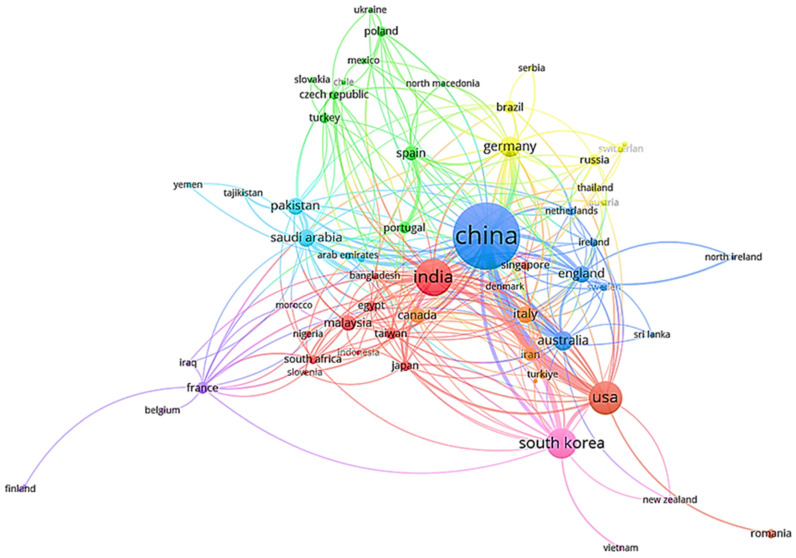
The co-authorship network encompasses countries engaged in collaborative research endeavors concerning polymer–graphene composites and functional materials. The size of the nodes indicates the number of publications produced by the country. The proximity of the two nodes indicates the strength of their co-authorship link, while the thickness of the connecting line indicates the strength of their cooperation. Articles: 60. Clusters: 9. Links: 286. Total link strength: 577.

Figure 2 shows how countries collaborate on publications indexed in Scopus and WoS that meet the search-key criteria reported in Table 1. This global co-authorship network shows the collaborative landscape exclusively within the design of high-efficiency electrochemical sensors through a network of nodes (countries) and their connections, where each connection (co-authorship link) reflects active participation in the development of these materials and technologies, including chemical selectivity, sensor stability, and surface functionalization, among others.

**Figure 3:**
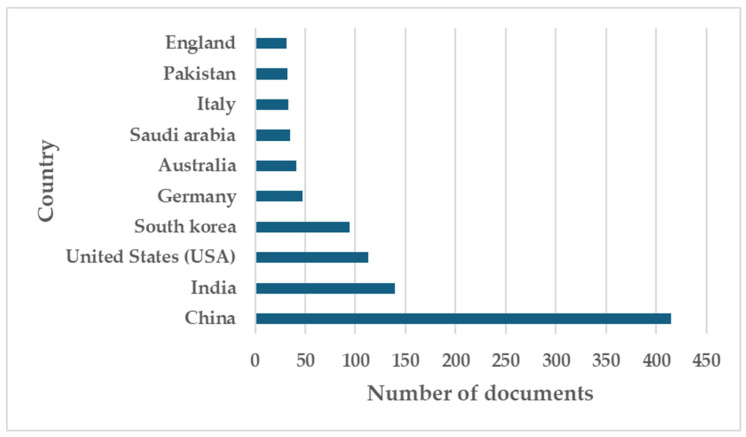
The top 10 countries for scientific production related to the use of polymer–graphene composites and functional materials with potential applications in electrochemical sensor design.

It is observed that China and the United States stand out in the network due to their enormous scientific productivity in graphene-based sensors, polymer–graphene composites, and hybrid platforms, among others, with a link strength of 39, followed by alliance with Australia, with a link strength of 22. Another important group is India, with a link strength of 88, 139 documents, and 32 links. The third group is South Korea with 94 documents, a total link strength of 53, and 21 links, while Germany has 47 documents, a total link strength of 71, and 28 links. These countries demonstrate strong cooperation, reflected in their proximity and link strength. Furthermore, it is important to note that European countries (in addition to the United States, Canada, and Australia) are the most active in cooperating with these global leaders in this field. Furthermore, the absence of some African countries (South Africa, Nigeria, among others), Asian countries (Japan, Hong Kong, Malaysia, Indonesia, Bangladesh, among others), and American countries (Brazil, Argentina, Mexico, Costa Rica, among others) demonstrates a lack of information and research on this topic in these continents, which could be due to insufficient investment in the necessary technologies. Consequently, the investment of government and institutional resources in research and development programs is imperative to promote such initiatives in these countries. Similarly, Figure 3 summarizes the 10 most influential countries in terms of scientific production oriented towards the development of materials and/or hybrid platforms based on graphene and conductive polymers, with China standing out with more than 400 documents in this field, followed by India, the United States, and South Korea. This trend is explained by extensive cooperation with other countries (as shown in Figure 2), which favors intergovernmental economic funding to support next-generation technologies based on graphene–polymer systems.

#### 3.1.1. Most Frequent Keywords Used to Report Research

**Figure 4:**
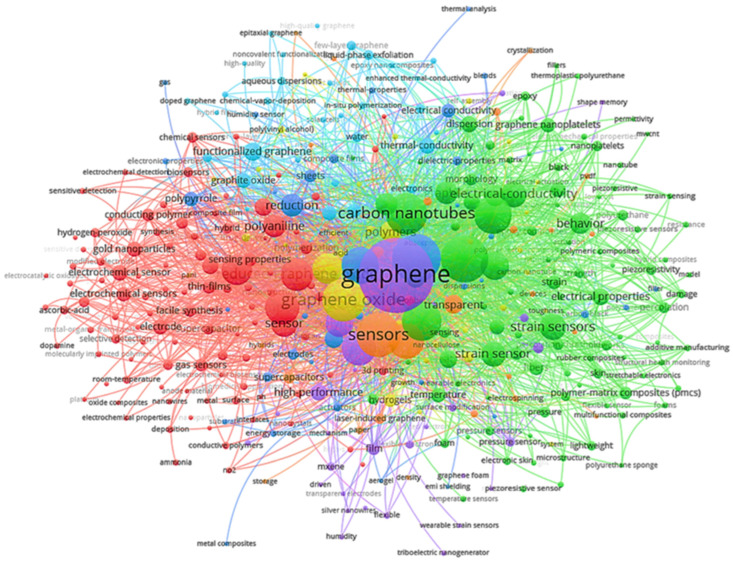
Co-occurrence network based on articles for terms. Curved lines of varying thickness, determined by co-occurrence, show related terms. The distance between nodes determines their relationship. Occurrence determines the size of the node.

Figure 4 presents a word cloud of keywords for this topic as a preliminary qualitative analysis. This was done to identify the most relevant words in all the reviewed articles on terms such as polymers and stimulus-responsive polymers, graphene, graphite and graphene compounds, electrochemical sensors and devices, properties, applications, and biodegradation, among others. This study revealed that the essential keywords in the topic were “graphene compounds,” “stimulus-responsive polymer,” and “stimulus-sensitive polymer,” among other prominent terms. This map reveals how knowledge is organized within the field: which core concepts are central, which thematic areas are interconnected, and which research pathways dominate in the field of advanced sensors based on hybrid materials.

The visual predominance of terms such as graphene, GO, carbon nanotubes, and sensors aligns with the designed search keywords and centers on materials with high electron mobility, enhanced conductivity, large surface area, and versatile chemical functionalization. Their coexistence with terms like graphene oxide, reduced graphene oxide, and carbon nanotubes reveals a spectrum of advanced carbon materials used to enhance sensor sensitivity, stability, and selectivity.

The construction of the keyword network entailed the selection of words that had been cited a minimum of ten times. These words were then distributed across 369 distinct terms, which were subsequently grouped into 7 clusters. The network thus exhibited a total link strength of 24,751. It resulted from eliminating overly generic keywords such as “human,” “non-human,” or identical terms. The first cluster (in red) covers sensors, electrodes, nanoparticles, and related topics. Terms such as “electrochemical sensor,” “thin-films,” “hybrids,” and “sensing properties” stand out as members of this cluster, whose main term is “reduced graphene oxide” with 224 links, a total link strength of 681, and an occurrence of 101.

The second group (green) relates to polymer composites and their properties. This cluster includes terms such as “carbon nanotubes” (280 bonds, a total bonding strength of 1119, and 150 occurrences) and “nanocomposites” (283 bonds, a total bonding strength of 1366, and 184 occurrences), highlighting the importance of recognizing these terms within the most cited articles worldwide, as a growing trend in the development of new technologies based on polymers and functional materials. The third group (blue) concerns applications of these materials, including terms such as “water,” “thermal conductivity,” and “electrical conductivity,” among others, which reflect the uses of composites and functional materials. The fourth group (yellow) is associated with materials and composites, featuring terms such as “graphene oxide,” “nanocomposite,” and “hydrogels,” among others. This indicates the relevance of research into functional materials and composites, including the development of highly specific electrochemical sensors.

#### 3.1.2. Which Are the Most Prominent Organizations in the Development and Scalability of These Materials?

**Figure 5:**
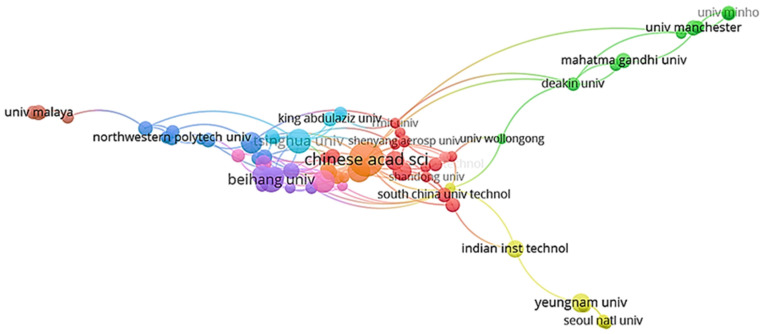
Co-citation Network Based on the Most Frequently Cited References (cited at least 30 times). The occurrence determines the term’s size. The relationship determines the distance between elements. Clusters: 10. Links: 147. Total Link Strength: 199.

Figure 5 shows the most cited and influential institutions in the literature and industry on polymers, graphene, and electrochemical sensors, establishing a highly sensitive indicator of the methodological, conceptual, and experimental impact of these institutions. Specifically, the node size and connection density indicate that the Chinese Academy of Sciences is the most-cited institution in collaborations and the center that produces the most highly influential research on graphene and GO, polymer composites, and the design of electrochemical sensors based on nanomaterials, among other areas.

**Figure 6:**
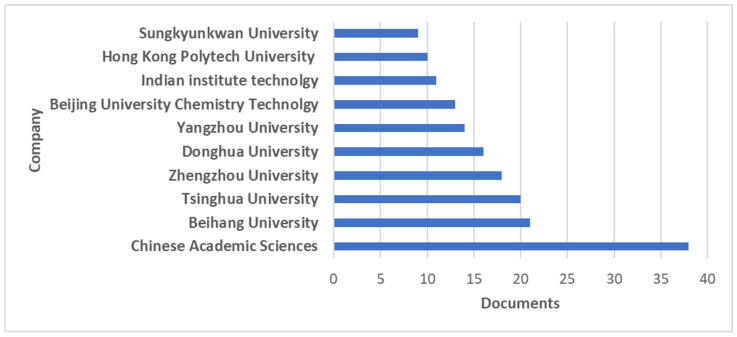
Leading institutions in the research and scalability of composites and functional materials.

Figure 6 shows the 10 leading institutions in scientific production focused on the design and synthesis of graphene–polymer hybrid materials, with productivity measured by the number of papers they have published. These industries and/or foundations are crucial to the development and expansion of innovative technologies across a wide range of applications, such as metal detection and the development of more efficient circuits and/or batteries with lower environmental impact. This trend is appropriate for strengthening governmental and institutional cooperation through economic incentives that promote research into more efficient materials for designing high-performance chemical sensors.

## 4. Literature Review: Structure, Chemistry, and Functionalization Strategies of Graphene

**Figure 7:**
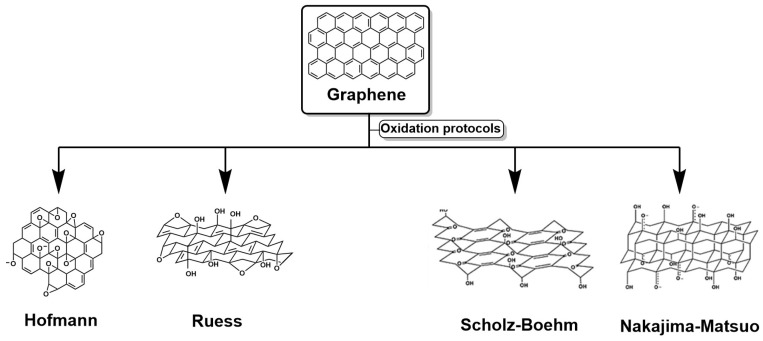
Proposed structural models for the GO. Adapted from Dreyer et al. [41]. From a chemical perspective, pristine graphene is relatively inert due to the absence of functional groups and the saturation of bonds in its basal plane. However, graphene chemistry can be activated through defect engineering and surface functionalization [42]. The controlled introduction of heteroatoms (N, B, S) alters the local electronic density, creating active sites for catalytic processes and modifying the Fermi energy. Similarly, partial oxidation of graphene produces GO, which is rich in epoxy (–O–), hydroxyl (–OH), carbonyls (–CO), and carboxyl groups (–COOH), increasing its hydrophilicity and facilitating dispersion in aqueous media and a broad network of bonded carbons with sp^2^ and sp^3^ hybridizations exhibiting hydrophobic domains [43]. Chemical or thermal reduction of GO yields reduced rGO, partially restoring π-conjugation and improving electrical conductivity [44,45].

The structures of graphene and oxidation models were introduced by Ruess, who employed classical methods for the oxidation of graphite and proposed that the basal skeleton could be reorganized into a fully sp^3^-hybridized topology [37]. Later, Hofmann and Holst proposed an architecture dominated by planes of sp^2^-hybridized carbon atoms, characteristic of graphite. This interpretation implied a deformed geometry based on cyclohexane-like units in which, periodically, a quarter of the rings harbored epoxide groups at positions 1 and 3. In contrast, position 4 was occupied by an—OH group (Figure 7), resulting in a network with a defined and stereochemically coherent repeating pattern [38]. In 1969, Scholz and Boehm offered a reinterpretation that completely abandoned epoxide and ether groups. Their proposal replaced these motifs with a wavy column composed of ordered quinoid units, generating a model with greater electronic continuity and without resorting to periodic oxygen functionalization [39]. This approach suggested a more dynamic behavior of the carbon network and broadened the spectrum of possible configurations for graphite oxide, as shown in Figure 7. Further development came from Nakajima and Matsuo, who approached the material’s structure from the perspective of fluorinated carbon compounds. Their analogy with poly(dicarbon monofluoride) allowed them to postulate that graphite oxide could be organized by a rigid lattice similar to that observed in second-stage graphite intercalation compounds. This parallelism provided a conceptual framework for integrating the layered nature of graphite and the modulating role of oxygen groups, without enforcing a strictly homogeneous geometry [40].

**Figure 8:**
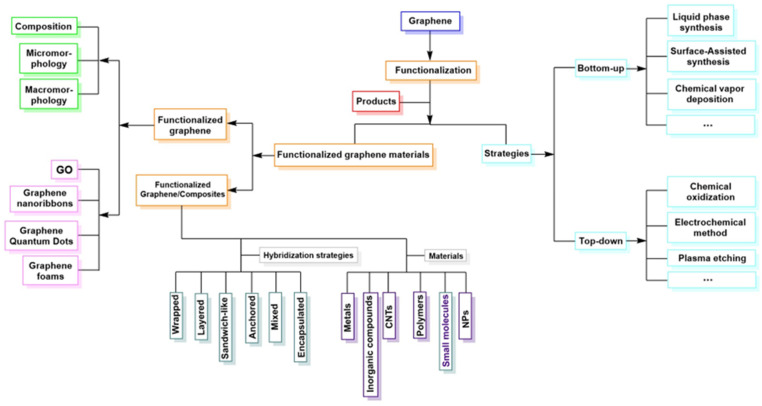
Diagram of graphene functionalization strategies. NPs: Nanoparticles, CNTs: Carbon nanotubes.

Figure 8 shows a schematic diagram that conceptually organizes graphene functionalization strategies hierarchically. In this sense, it can be observed that graphene functionalization strategies integrate specific structural control to obtain advanced properties and morphological diversity. These materials can be hybridized with nanoparticles, polymers, inorganic compounds, and nanotubes, among others, resulting in high-performance materials.

Among the most widely used methods for synthesizing GO from graphite, the Hummers method stands out as a classic procedure for its efficiency in chemical oxidation. This method employs a concentrated mixture of sulfuric acid (H_2_SO_4_), sodium nitrate (NaNO_3_), and potassium permanganate (KMnO_4_) at a controlled temperature (typically 35–45 °C) to prevent violent reactions. Although widely used, it generates toxic byproducts, including NO_2_ and N_2_O_4_, which pose environmental and safety risks [46]. Modified variants of the Hummers method incorporating TiO_2_ aim to improve oxidative efficiency and reduce the formation of harmful gases [47]. TiO_2_ acts as a photocatalytic agent, enabling more homogeneous oxidation and reducing reaction time. These modifications also promote the production of GO sheets with fewer structural defects.

Additionally, improvements to the Hummers method have been reported that eliminate the use of NaNO_3_, thereby reducing nitrogen oxide emissions and increasing process safety. These modifications also optimize the KMnO_4_/graphite ratio and employ thermal pretreatments or sonication to facilitate exfoliation [48,49,50]. Such improvements enable the production of GO with a higher degree of oxidation and better dispersion in aqueous media [51,52,53,54], and represent a more sustainable alternative. This approach uses aqueous electrolytes (such as sulfate or chloride solutions) and applies a controlled electric potential to induce the separation of graphite layers and their simultaneous oxidation. Among its advantages are the absence of highly corrosive reagents and the ability to adjust the oxidation degree by varying the applied voltage. However, large-scale production remains a challenge due to the need for specialized equipment [55,56,57].

### 4.1. Defects and Surface Modification of Graphene: Implications in Advanced Electrochemical Systems

**Figure 9:**
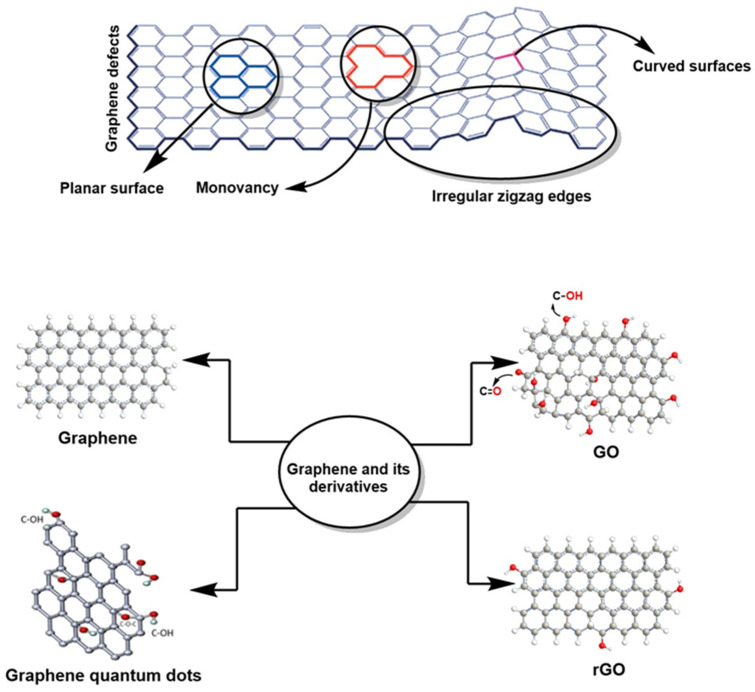
Molecular structure of traditional graphene-based materials.

Graphene is a monolayer of carbon atoms arranged in a two-dimensional lattice with hexagonal symmetry and an estimated surface area of 2630 m^2^g^−1^ (as shown in Figure 9). These characteristics make graphene an ideal material for a wide range of electrocatalytic reactions, and its high surface area facilitates the uniform dispersion of other active materials, promoting the formation of electroactive centers for various electrochemical conversions. This configuration imparts exceptional properties to the material, including an electron mobility of up to 200,000 cm^2^ V^−1^ s^−1^ and a thermal conductivity exceeding 3000 W·m^−1^ K^−1^, placing it at the forefront of advanced materials [58,59,60,61]. Figure 9 shows the typical structures of graphene and its derivatives, as well as structural imperfections that influence their interactions with and reactivity toward other systems or molecules, whether due to disruptions in electron flow, electrical conductivity, hybridization, etc. However, several studies have reported significant progress in overcoming these limitations by exfoliating GOs produced using the Hummer method. For example, the development of hybrid electrochemical biosensors with improved flexibility, employing GO coatings doped with Au and Pt nanoparticles for monitoring nitrogen oxides, is achieved by ligand exchange between Au@Pt nanoparticles and 2,2–dithiobios[1–(2–bromo–2–methylpropionoxy)]ethane (DTBE) to create a metallic film, resulting in the modified Au@Pt–rGO electrode [62]. This electrode, with improved flexibility and bimetallic nanoparticle doping, exhibited high sensitivity, a wide linear range from 400 nM to 673.9 μM, and a low detection limit of 100 nM for amperometric detection of nitrogen oxides.

Several articles have reported various materials derived from graphene: two-dimensional materials such as GO and three-dimensional materials such as graphene hydrogels and/or foams [63]. GO exhibits specific structural characteristics that can either favor or affect various materials. For example, this material has –COOH functional groups at its edges, which disrupts or affects electrical conductivity due to the disruption of the sp^2^ bond network, resulting in a conductivity of 0.02–0.07 S m^−1^; this is low compared to graphene, which exhibits a conductivity of 1 × 10^8^ S m^−1^ [64]. However, it is possible to recover the conductivity of GO by restoring the sp^2^ lattice at over 3000 S cm^−1^ [65], which is achieved through doping with hydrogen atoms as well as nitrogen, sulfur, metal oxides, nanoparticles, and conducting polymers. This results in hybrid materials with electrochemical performance superior to that of various traditional metal electrodes due to increased charge-carrying capacity (i.e., a material’s ability to donate or accept electrons) [66].

Furthermore, the flexibility and surface area of these electrodes are fundamental characteristics for achieving improved properties. For example, the inclusion of graphene-conjugated gold nanorods (AuNRs) for electrochemical sensing has been reported [67,68]. For example, silicate sol–gel matrices functionalized with amines such as N1-[3-(trimethoxysilyl)propyl]diamine (TPDT) loaded onto rGO and AuNR composites (rGO-Au-TPDT NRs) have also been reported [62]. For instance, silicate sol–gel matrices functionalized with amines such as N1-[3-(trimethoxysilyl)propyl]diamine (TPDT) loaded onto rGO and AuNR compounds (rGO-Au-TPDT NRs) have been reported, exhibiting synergistic catalytic effects in rGO-Au-TPDT NRs, with a detection range between 10 and 140 nM, and a detection limit of 6.5 nM for nitrogen monoxide detection [69]. In this context, the electrochemical properties of these materials can be exploited through surface modifications that leverage the reactivity of carbonyl groups present in GO, which are fundamental for electrochemical detection and high-efficiency molecular recognition. Consequently, these strategies enable the design of materials with enhanced properties for applications in energy, electronics, and biotechnology, highlighting the sustained growth of this versatile family of materials [67,70].

#### 4.1.1. Chemical Functionalization of Graphene Using Carboxyl Groups: Fundamentals and Structural Control

The presence of –CO and –COOH functional groups at the edges of the GO plays a fundamental role in the chemical modification of graphene, according to the Lerf–Klinowski model, as does the presence of –O– and –OH groups on the basal planes, since these provide reactive sites for the formation of covalent bonds and the incorporation of new functionalities [71]. These groups favor orthogonal reactions; however, the selectivity of these chemical transformations remains a significant challenge [72]. That is, in many cases, a single reaction can simultaneously involve multiple surface functionalities, a situation exacerbated by the wide structural and compositional heterogeneity of GO [73]. This complexity makes the isolation and rigorous characterization of the reaction products very difficult in practice.

Covalent coupling reactions on graphene oxide often require prior activation of the carboxylic acid group to increase its reactivity towards nucleophilic species. Activating agents commonly used for this purpose include thionyl chloride (SOCl_2_) [74,75], 1-ethyl-3-(3-dimethylaminopropyl)-carbodiimide (EDC) [76], *N*,*N*′-dicyclohexylcarbodiimide (DCC) [77], or 2-(7-aza-1H-benzotriazol-1—yl)-1,1,3,3-tetramethyluronium hexafluorophosphate (HATU) [78]. These reagents convert these groups into highly reactive intermediates, facilitating subsequent functionalization steps.

**Figure 10:**
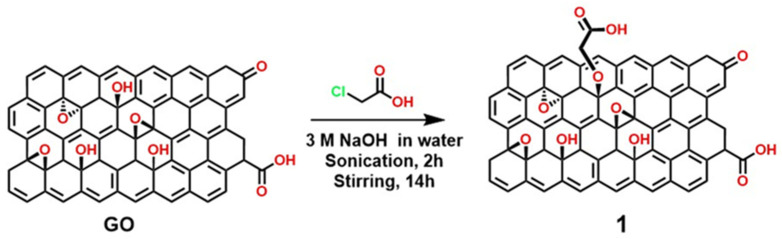
Carboxylation reaction on –OH groups present in the GO.

For example, Figure 10 illustrates the functionalization of GO via carboxylation using the Hummers method with concentrated NaOH and chloroacetic acid to obtain material 1, which exhibited greater thermal stability (150–200 °C). It is important to note that the carboxylation reactions shown in Figure 10 occur only on –OH groups, while reactions involving epoxy rings with NaOH establish an equilibrium, and the C–O groups may close again to form a ring depending on the pH conditions [79,80].

Carboxylation of GO with chloroacetic acid in an alkaline medium is a classical strategy to increase the surface density of –CO groups. However, this procedure often involves partial reduction phenomena that are not always documented, thereby significantly altering the material’s surface chemistry and electronic properties. Reduction under strongly alkaline conditions, even at room temperature, has been reported as an effective method for obtaining rGO, suggesting that functionalization and reduction may occur simultaneously, generating hybrids with unique characteristics [81,82,83,84]. In electroanalytical terms, the higher density of –COOH groups favors the EDC/NHS anchoring of bioreceptors and hydrophilic polymers (e.g., chitosan/PEG), improving selectivity and reproducibility in complex matrices. However, partial loss of π conjugation can increase the overpotential and decrease sensitivity; partial rGO and heteroatomic doping mitigate this functional effect, combining customization with efficient electron transfer.

To explore this synergy, a combination of epoxy ring opening with controlled carboxylation has been proposed, followed by an amidation step using polymeric derivatives such as O-(2-aminoethyl)-O′-[2-(Boc-amino)ethyl]decaethylene glycol (BocNH-PEG_10_-NH_2_) (material 2), adding chloroacetic acid in the presence of 3M NaOH under sonication to generate material 3, which exhibited greater thermal stability compared to material 2, likely due to deoxygenation under strongly basic conditions. After the amidation reaction, additional polyethylene glycol (PEG) chains were grafted onto the GO surface, yielding material 4 with improved thermal properties (200–400 °C) [72].

**Figure 11:**
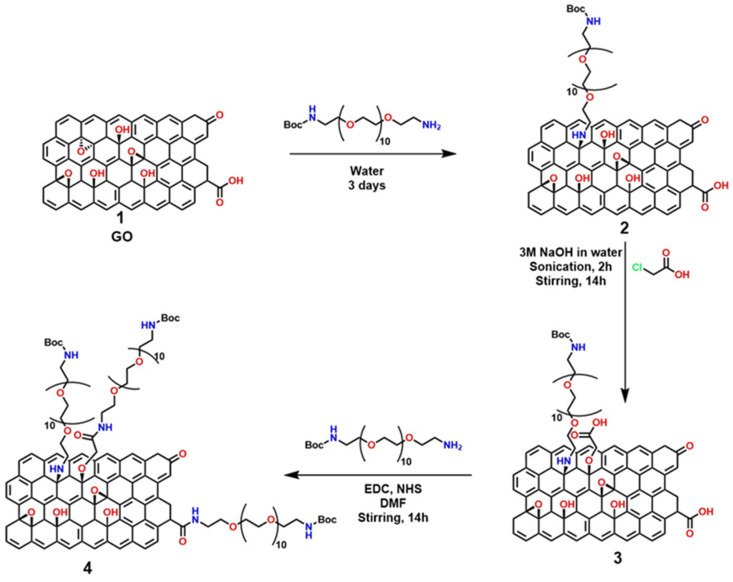
Mechanism of double functionalization of GO by combining the epoxide ring opening reaction in carboxylation reactions.

This approach, activated through 1-ethyl-3-(3-dimethylaminopropyl) carbodiimide (EDC)/N-hydroxysuccinimide (NHS) systems, enables the introduction of two types of functionalities: carboxyl groups and Boc-protected polymer chains, which provide controlled hydrophobicity and sites for subsequent modifications, as illustrated in Figure 11. Dual functionalization not only enhances GO’s chemical versatility but also opens new opportunities in nanobiotechnology to develop platforms for biomolecule immobilization and controlled drug release [85]. Furthermore, its electronic properties benefit from improved dispersion within polymer matrices and conductivity tuning [86].

#### 4.1.2. Advanced Functionalization of the GO Through “Click” Reactions and Covalent Coupling

**Figure 12:**
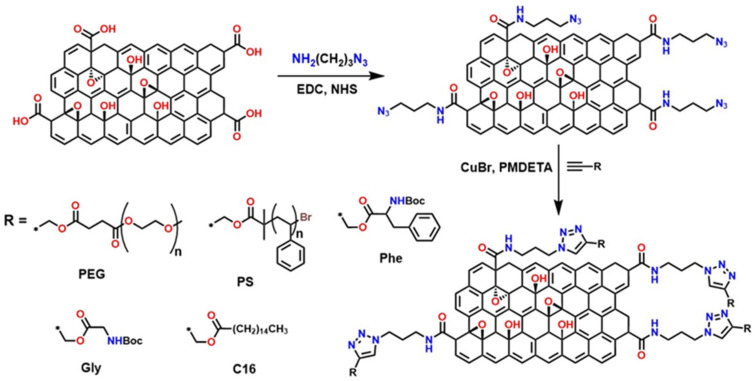
Functionalization of GO nanosheets through click-type reactions for polymer insertion. The symbol (*) indicates the reactive site, the anchor on the polymer chain or small molecule (R group) that participates in the coupling reaction with the azide-functionalized graphene oxide.

Several strategies have been reported that exploit the high reactivity of the –COOH groups present in GO to introduce azide groups (–N_3_) through EDC-activated condensation reactions. This initial step generates GO—N_3_, a key intermediate that enables subsequent functionalization via copper-catalyzed azide–alkyne cycloaddition (CuAAC), commonly known as click chemistry, as illustrated in Figure 12. This methodology is highly efficient, regioselective, and compatible with mild conditions, making it an ideal tool for modifying nanomaterials. Once azide groups are incorporated, GO can react with various compounds containing alkyne termini, allowing the synthesis of derivatives with specific properties: GO—PEG (enhances dispersion in aqueous media and biocompatibility, ideal for biomedical applications) [87], GO—PS (improves compatibility with hydrophobic polymer matrices, useful in nanocomposites) [88], GO—C16 (C16—palmitic acid modification provides hydrophobicity and thermal stability, favoring applications in coatings) [89], and GO—Gly (Gly—glycine) and GO—Phe (Phe—phenylalanine), which introduce amino acids that can offer active sites for bioconjugation, sensors, or enzymatic catalysis [90].

**Figure 13:**
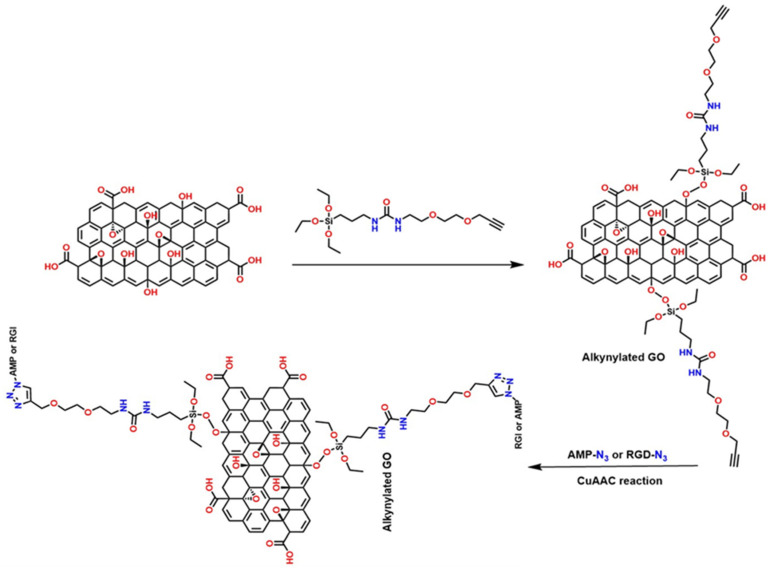
Schematic of peptide insertion into GO via click reactions.

Similarly, the incorporation of the Arg—Gly—Asp peptide sequence (arginine—glycine—aspartic acid) and adenosine monophosphate (a nucleotide composed of adenine, ribose, and a phosphate group) into functionalized GO via click chemistry has been reported, as shown in Figure 13. The preparation of functionalized materials such as GO-c-RGD (Arg—Gly—Asp peptide) and GO—c—AMP (adenosine monophosphate) relies on the creation of specific reactive sites on GO nanosheets to enable the click reaction [91,92,93,94].

## 5. Polymer–Graphene Hybrid Architectures

Hybrid structures based on GO and various polymers have emerged as one of the most versatile strategies for exploiting GO’s intrinsic properties while overcoming some of its structural and processability limitations compared to conventional materials. From a structural perspective, incorporating GO into polymer systems can lead to hierarchical architectures in which two-dimensional sheets act as nanometric reinforcements or as active functional elements. For example, when polyvinylpyrrolidone (PVP) grafts are mixed with GO under agitation, PVP adsorbs onto the material’s surface via hydrophobic interactions and Van der Waals forces, forming a protective layer that prevents sheet aggregation. This coating not only improves colloidal dispersion in aqueous media but also provides chemical and mechanical stability, essential for applications in biological and electrochemical environments [95].

During the process, the PVP–graphene interaction is non-covalent, meaning that the electronic structure of graphene remains virtually intact, preserving its conductive properties [96]. This feature is advantageous for applications that require high conductivity, such as electrocatalysts for the reduction of oxygen (O_2_) and hydrogen peroxide (H_2_O_2_). Moreover, the ease of redispersion via ultrasonication makes PVP—GO an attractive material for scalable, cost-effective processes. However, its stability may be compromised in harsh media due to the physical nature of the interaction [96].

In contrast to physical adsorption, covalent functionalization offers greater chemical robustness and functional versatility. The procedure described by Batool et al. [97] begins with the silanization of GO using 3-glycidoxypropyltrimethoxysilane, which reacts with hydroxyl groups present on the GO surface. This step generates Si–O–C bonds, creating stable anchoring points for the incorporation of more complex molecules [98]. Subsequently, a fourth-generation PAMAM dendrimer with an ethylenediamine core is introduced, providing a high density of amino groups on the graphene surface. This modification not only improves hydrophilicity and aqueous dispersion but also supplies reactive sites for the immobilization of biomolecules such as enzymes, antibodies, or peptides. Furthermore, PAMAM treatment partially reduces GO, thereby increasing its electrical conductivity, a key factor for applications in electrochemical biosensors [98].

**Figure 14:**
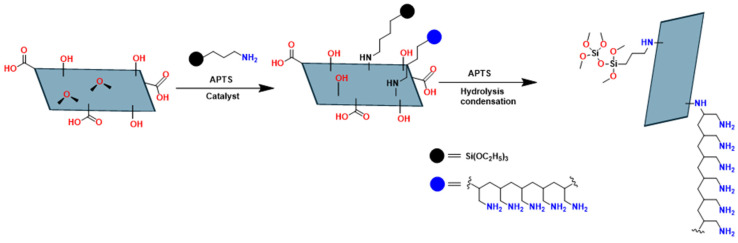
Covalent functionalization of GO using epoxide groups for the grafting of silane and poly(allylamine) groups.

Additionally, the –O– functional groups present in GO are of great importance for stabilizing solid dispersions of carbon-based materials at the nanoscale. In this context, Yang et al. explored the reactivity of epoxides as anchor points for the covalent modification of graphene oxide platelets using 3-aminopropyltriethoxysilane (APTS) (Figure 14) [99]. The APTS silane chains are grafted onto the surface of the material through an SN_2_-type nucleophilic displacement process, in which the amino groups open the epoxide ring and generate a stable covalent bond between the two species, as well as functionalization with poly(allylamine) (an aliphatic main-chain polymer with amine groups), which provides versatility to materials due to its great capacity to coordinate metals.

### Nanocomposites Based on Conductive Polymers and GO

Polymers such as chitosan (a copolymer composed of N-acetylglucosamine units (2-(acetylamino)-2-deoxy-D-glucopyranose) and glucosamine (2-deoxy-2-amino-D-glucopyranose)) are polycationic polymers derived from chitin, characterized by high water permeability and remarkable susceptibility to chemical modifications due to the presence of reactive amino (–NH_2_) and hydroxyl (–OH) groups. These functionalities enable strong interactions with carbon-based surfaces and facilitate the formation of covalent or non-covalent bonds [100].

For instance, Han et al. reported the synthesis of graphene functionalized with chitosan through a combined mixing method and in situ chemical reduction. During this process, chitosan molecules intercalate between graphene layers, acting as both a dispersant and a stabilizer, thereby preventing aggregation and improving the material’s processability [101]. The chitosan–GO nanocomposite reported by Han et al. exhibited high sensitivity and selectivity for the electrochemical detection of biomolecules, including ascorbic acid, dopamine, and uric acid, making it an ideal candidate for biosensor applications. Moreover, the amino groups present in chitosan provide numerous active sites for further functionalization, enabling the immobilization of enzymes, antibodies, or other biomolecules [102,103]. This feature broadens its applicability in biomedical diagnostics and bioelectronic devices.

Similarly, polyethyleneimine (PEI) is another polymer rich in amino groups, capable of acting as a reducing agent to convert GO into rGO while covalently incorporating onto its surface [104]. The synthesis of PEI—rGO via reflux results in a material with a positive charge and excellent adsorption capacity. This modification enhances electrostatic interactions with negatively charged analytes, resulting in superior detection of phenolic compounds such as gallic acid. Furthermore, the high density of amino groups in PEI enables bioconjugation with proteins and nucleic acids, expanding its use in biosensors and biofunctional platforms [104].

Additionally, poly(allylamine hydrochloride) (PAH) is a weak cationic polyelectrolyte with numerous ionizable amino groups, fully protonated in neutral and acidic solutions [105]. Dispersion of GO in an aqueous PAH solution via ultrasonication yields a stable suspension suitable for fabricating modified electrodes. The presence of PAH not only stabilizes the material but also promotes electrostatic interactions with negatively charged biomolecules, such as nicotinamide adenine dinucleotide (NADH), thereby enhancing the electrochemical response [105].

On the other hand, the development of an overoxidized polypyrrole (PPy)—GO nanocomposite via electrochemical synthesis has been reported, employing cyclic voltammetry in a solution containing pyrrole monomer, GO, and LiClO_4_ as the electrolyte. This method enables precise control over polymer growth on the GO surface, generating a highly porous nanostructure. The porosity significantly increases the active surface area, thereby improving adsorption capacity and charge transfer [106]. The PPy—GO nanocomposite exhibits strong π–π interactions with nitrogenous bases, such as adenine and guanine, facilitating their adsorption and enabling high-sensitivity electrochemical detection. This property makes it a promising material for nucleic acid biosensors, where selectivity and electrocatalytic capability are essential [106].

Other polymers, such as poly(3,4-ethylenedioxythiophene) (PEDOT), exhibit conductive properties along with high chemical and electrical stability. For instance, electropolymerization of the EDOT monomer onto rGO, followed by electrodeposition of cobalt nanoparticles (CoNPs), results in a fibrillar structure of the PEDOT—rGO nanocomposite, which plays a crucial role in the nucleation and homogeneous distribution of CoNPs, thereby enhancing the material’s catalytic activity and stability. This type of hybrid is particularly useful as an electrocatalyst for redox reactions and in advanced sensors [107,108]. Subsequent modification via covalent conjugation to aptamers enables highly sensitive and selective detection of dopamine, demonstrating the potential of these materials for molecular biosensors [109]. In this context, the combination of conductive polymers with GO provides a versatile platform for biomolecule immobilization, leveraging the synergy among electrical conductivity, high surface area, and chemical functionality. For example, numerous monomers have been explored for the electrochemical synthesis of polymer–GO nanocomposites, following similar strategies. Among them are aromatic and heterocyclic derivatives such as aminobenzene sulfonic acid [110], xanthurenic acid [111], indole-5-carboxylic acid [112], sulfosalicylic acid [113], 2,6-pyridinedicarboxylic acid [114], carbazole [115], imidazole [116], melamine [117], quercetin [118], and methylthiophene [119], among others.

## 6. Electrochemical Sensing Applications

### 6.1. Voltammetric Sensors Derived from Functionalized Graphene

Electrochemical applications, such as voltammetric methods, are the most widely used transduction techniques for designing electrochemical sensors due to their high sensitivity, speed, and ability to characterize redox processes at the electrode surface. These techniques are based on measuring the current generated by the oxidation or reduction of the analyte at a modified electrode during a controlled potential sweep in the presence of a supporting electrolyte [120,121].

**Figure 15:**
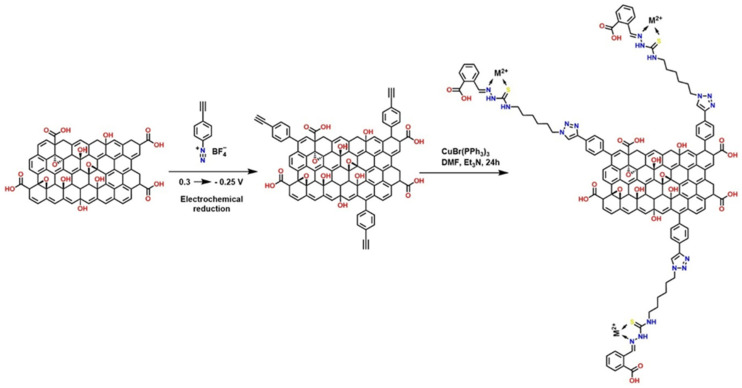
Electrode modification via click chemistry.

A significant advantage of voltammetric approaches is that, in addition to providing accurate quantitative analysis, they provide detailed insights into electron-transfer kinetics and mechanisms, as well as interactions between the analyte and the electrode material. This diagnostic capability is essential for optimizing sensor architecture and understanding phenomena such as adsorption, diffusion, and the formation of intermediate products [121,122]. The incorporation of graphene-based nanocomposites into voltammetric electrodes has revolutionized sensor performance. Thanks to its high electrical conductivity, large surface area, and capacity for chemical functionalization, graphene enhances sensitivity, selectivity, and detection limits. Moreover, its two-dimensional structure facilitates the immobilization of biomolecules and catalysts, boosting the electrochemical response, as shown in Figure 15. In voltammetry, rGO + AuNPs/PEDOT composites shift the oxidation potential and increase the analytical slope, enabling µM LOD for NO/H_2_O_2_; however, their cost and sensitivity to poisoning justify alternatives such as NiO/Cu_2_O over GO/rGO, which maintain good metrics in alkaline media.

Voltammetric sensors based on graphene nanocomposites have played a prominent role in electrochemical detection due to their high conductivity, large surface area, and ability to facilitate electron transfer. These devices not only enable high sensitivity but also allow simultaneous determinations of analytes that, under conventional conditions, could cause interferences [123].

Among the most employed voltammetric techniques for this purpose are linear sweep voltammetry (LSV), differential pulse voltammetry (DPV), square-wave voltammetry (SWV), and stripping voltammetry (SV). Each of these methodologies exhibits specific characteristics that make it suitable for quantifying different chemical species [124]. For instance, they have been successfully applied in the detection of metal ions, low-molecular-weight organic molecules, and even complex biomolecules such as proteins, highlighting their versatility in analytical and biomedical settings [125,126,127]. Table 2 reports several graphene-based functionalized electrodes for detecting emerging contaminants, which exhibit low detection limits, an ideal feature for environmental monitoring.

### 6.2. Amperometric Sensors Derived from Functionalized Graphene

In amperometry, the current generated in the cell is recorded as a function of time after applying a constant potential. This technique enables the sequential addition of analytes and is particularly useful for electroactive compounds that exhibit time-dependent redox processes. Amperometric sensors have been widely used for sensitive detection of species such as hydrogen peroxide (H_2_O_2_) [134,135,136] and glucose [137,138,139], among others, as reported in Table 3.

H_2_O_2_, a secondary product of numerous biochemical reactions, plays essential physiological roles in biological systems, thereby justifying interest in its precise quantification. Several studies have explored electrodes modified with nanomaterials to enhance the amperometric response toward this compound. For example, Noor et al. developed an electrode based on silver nanoparticles supported on GO, achieving excellent electrocatalytic activity toward H_2_O_2_. The sensor exhibited an I–T (current vs. time) curve with a linear range from 100 μM to 11 mM and a detection limit of 28.3 μM [135]. In another study, Zhang et al. developed a more sensitive sensor for H_2_O_2_ reduction based on rGO–platinum nanocomposites. This device operated at a significantly low potential (0.08 V vs. Ag/AgCl), with a wide linear range (0.5 μM–3.475 mM), high sensitivity (~460 mA·M^−1^·cm^−2^), and a detection limit of only 0.2 μM, outperforming electrodes modified solely with PtNP or graphene. Furthermore, its application extended to monitoring H_2_O_2_ release in living cells [140]. Additionally, Ensafi et al. investigated H_2_O_2_ oxidation using a glassy carbon electrode coated with Nafion and a GO—Co_3_O_4_ nanocomposite. Under amperometric conditions (0.76 V vs. Ag/AgCl), the sensor showed a detection limit of 0.3 μM and a linear response spanning four orders of magnitude, confirming its potential for demanding analytical applications [141].

The studies reported in Table 3 demonstrate that integrating nanomaterials with GO and rGO has transformed the design of electrochemical sensors, delivering remarkable improvements in sensitivity, stability, and response speed. Each approach combines unique properties: from graphene’s high conductivity to the catalytic activity of metals and metal oxides. For instance, sensors based on AuNPs-ATPGO and Pt–PANI–GE stand out for their excellent precision and reproducibility, making them ideal for pharmaceutical applications. However, their synthesis involves multiple steps and the use of noble metals, which increases cost [142,143]. Similarly, NiO–GR and Cu_2_O–rGO systems offer more cost-effective alternatives with good performance, although they require alkaline conditions that limit their direct use in biological fluids [138,144,145].

**Table 3:** Amperometric sensors based on GO functionalized with nanomaterials.

Modified Electro Type	Analytes Detected	Work Potential	Detection Limit (μM)	Advantages/Disadvantages	Ref.
Pt-PANI-GR/GCE	NO_2_^−^	0.75 V (vs. SCE)	0.13	Advantages: High sensitivity and good analytical performance, tNPs (~5 nm) uniformly dispersed on PANI-graphene, with clear improvements in electron transfer (EIS) and increased oxidation currents compared to GCE, GO/GCE, and PANI-GE/GCE. Disadvantages: The detection limit is adequate for high nitrite levels but less competitive than other sensors that reach the nM range; it might require pre-concentration for trace levels in strict environmental matrices.	[146]
GR/Au/GCE	NO	0.8 V (vs. SCE)	0.018	Advantages: Negative shift of the oxidation potential (~220 mV) compared to bare GCE, thanks to the synergy between graphene (high area and conductivity) and AuNPs (electrocatalysts). Furthermore, it acts as a barrier to anions and proteins, allowing free diffusion of NO. Minimal interference with ascorbic acid, uric acid, L-arginine, and xanthine (<4 μM). Disadvantages: It requires controlled electrodeposition of AuNPs, graphene coating, and Nafion, which involves several steps and specialized reagents. Furthermore, it exhibits strong dependence on strict conditions: optimal pH of 7.0 (PB) and an applied potential of +0.80 V vs. SCE; outside these conditions, the response may decrease.	[147]
GO-Au/PDAN-EDTA/GCE	Dopamine	0.3 V (vs. Ag/AgCl)	0.005	Advantages: It exhibits high selectivity in the presence of common interferents. That is, the GO/AuNPs/pDAN-EDTA film carries a negative charge (due to EDTA) that repels ascorbic acid and uric acid (anionic at neutral pH) while attracting dopamine (cationic), thereby preventing signal overlap and electrode fouling. Disadvantages: Requires electropolymerization synthesis of pDAN and activation/assembly with EDTA (EDC/NHS); several steps increase time and variability if not standardized.	[148]
Co_3_O_4_-GR/GCE	Tyrosine	0.95 V (vs. SCE)	0.001	Advantages: It exhibits acceptable selectivity and validation in real samples. That is, signal separation against AA and DA in DPV (well-resolved peaks at pH 2.0). In addition, it provides better film dispersion and adhesion than drop-casting, favoring uniformity and electrochemical stability. Disadvantages: It is susceptible to interference from metallic cations such as Fe^3+^ and Al^3+^, which reduce the current (ratios of 0.71 and 0.80), necessitating ionic composition control or pretreatment in complex samples.	[149]
rGO/GCE	Promethazine	0.78 V(vs. Ag/AgCl)	0.199	Advantages: Its manufacture is simple and environmentally friendly, achieved by electrochemically reducing GO in an acetic acid medium (pH 5.0), avoiding the use of toxic reducing agents and high temperatures. Disadvantages: Requires strongly acidic conditions, with an optimum at 0.10 mol·L^−1^ H_2_SO_4_ (pH ≈ 2.0); at neutral pH, the signal drops drastically (42% after 11 cycles), limiting applications in physiological matrices.	[150]
GO-Ag/GCE	Glucose	0.6 V (vs. SCE)	4	Advantages: Increased anodic current (~1.5 times greater than NiO/GCE) and reduced overpotential for glucose oxidation, thanks to the high conductivity and active area of the GO. It exhibits selectivity against common interferents such as sucrose, fructose, citric acid, acetic acid, ethanol, and ethyl acetate, which cause interference <5%; ascorbic acid affects glucose only at concentrations comparable to glucose. Disadvantages: Requires reduced graphene synthesis (GO → GR with ascorbic acid), pulsed electrodeposition of Ni, and potential cycling to form NiO; several steps can affect reproducibility if not controlled.	[138]
PB@GR/GCE	H_2_O_2_	−0.05 V(vs. SCE)	0.005	Advantages: Reduction of H_2_O_2_ to ~0.43 V (vs. SCE), lower than in conventional PB (~0.60 V), which reduces interference from metabolites such as glucose and ascorbic acid. No significant current loss after 30 cycles, thanks to the strong PB-graphene interaction. Disadvantages: It requires hydrogel formation, solvent exchange for a week, and supercritical drying, which limits industrial scalability.	[136]
rGO-Cu_2_O/GCE	Glucose	0.6 V (vs. SCE)	0.05	Advantages: It exhibits synergy with the Cu_2_O–RGOs system. Furthermore, the porosity of Cu_2_O (specific surface area ≈ 22.5 m^2^·g^−1^) and the high conductivity of RGOs generate a greater number of active sites and enable efficient electron transfer. Disadvantages: It requires 50 mM KOH to activate electrocatalysis, which limits direct applications in physiological fluids without pH adjustment. Furthermore, it requires optimal conditions at 0.6 V vs. SCE, which can favor the oxidation of interferents in complex matrices.	[139]

### 6.3. Potentiometric Sensors Derived from Functionalized Graphene

Potentiometric sensors measure the potential difference between a reference electrode and a working electrode, whose potential varies with analyte concentration according to the Nernst equation. This approach is primarily applied to ionic species, although it can also be used in some cases for neutral molecules [151,152]. A notable example is the work by Ping et al., who developed a flexible potentiometric sensor using graphene paper as a conductive substrate and ion-to-electron transducer. This material enabled the construction of three integrated devices combining ion-selective electrodes with graphene-based reference electrodes, achieving performance comparable to conventional systems and excellent mechanical flexibility [153].

On the other hand, Abraham et al. designed a sensor for Pb^2+^ detection using an electrode modified with benzene 1,2-bis(N′-benzoylthioureido) (BBTB)–rGO. The device exhibited a Nernstian slope of 30.37 ± 0.62 mV/decade within a concentration range of 6.31 × 10^−8^ to 3.98 × 10^−2^ M, maintaining stability across a wide pH interval (4–8) [154]. Similarly, Poursaberi et al. introduced a fluoride-selective electrode based on metalloporphyrin–GO grafts, in which the incorporation of graphene improved critical parameters, including dynamic range, detection limit, response time, and stability. This sensor exhibited a linear response over the range 5.0 × 10^−7^ to 5.0 × 10^−1^ M, with a Nernstian slope of −58.3 mV/decade and a response time of only 20 s [155].

### 6.4. Electrochemiluminescence Sensors Derived from Functionalized Graphene

Electrochemiluminescence is an electrochemically induced optical phenomenon that originates from the relaxation of excited species generated at the electrode surface. One of its main advantages is that it does not require an external light source, eliminating interference from light scattering and fluorescent impurities and significantly increasing the method’s sensitivity. Moreover, electrochemiluminescence offers high specificity, arising from the interaction between the luminophore and the co-reactant, and remarkable selectivity, as the excited states can be controlled by modulating the applied potential [156,157].

Graphene-based materials have established themselves as ideal supports for luminophores due to their high conductivity, abundant active sites, and excellent electrochemical properties [158]. Among the most widely used electrochemiluminescent (ECL) reagents, the Ru(bpy)_3_^2+^ complex stands out for its strong luminescence and good electroactivity. This complex can be effectively immobilized on graphene via π–π interactions, thereby enhancing electron transfer and system stability. For example, Gu et al. developed a solid-state ECL sensor via screen-printing on paper, incorporating a mixture of Ru(bpy)_3_^2+^/PSS-GN/carbon paste. The device exhibited excellent sensitivity for detecting tripropylamine (TPA), achieving a detection limit of 5.0 nM. Complementarily, Li et al. designed an ECL biosensor based on Ru(bpy)_3_^2+^ nanowires (RuNWs) immobilized on a glassy carbon electrode modified with composite films of rGO–Nafion [159]. The incorporation of rGO enhanced the electrochemical signal and, consequently, the ECL intensity. Using TPA as a co-reactant, the sensor achieved a wide linear range (1.0 × 10^−12^–1.0 × 10^−5^ M) and an extremely low detection limit (3.1 × 10^−13^ M) for dopamine, highlighting its potential for biomedical applications [160].

Another notable example is the work by Wang et al., who developed an ECL sensor using CdSe quantum dots (QDs) incorporated into a porous GO–chitin (GO–CHIT) matrix. This structure not only enabled higher QD loading and a broad reaction interface but also facilitated the diffusion of the co-reactant K_2_S_2_O_8_ through the membrane, resulting in an intense ECL signal, good biocompatibility, and prolonged stability. The device was successfully applied to the detection of cytochrome C (Cyt C) [161]. Similarly, Lv et al. designed an ECL sensor based on graphene oxide intercalated with polypyrrole (NH_2_–GO) and Ag_2_Se@CdSe nanoneedles. The high surface area of NH_2_–GO enabled greater loading of nanoneedles, thereby increasing ECL intensity and improving system sensitivity. This sensor was employed to detect the tumor marker CA72-4 in the presence of K_2_S_2_O_8_, showing a linear relationship over the range of 10^−4^ to 20 U·mL^−1^ and a detection limit of 2.1 × 10^−5^ U·mL^−1^ [162]. Likewise, Xin et al. developed ECL cytosensors based on a ternary nanocomposite of hemin–rGO–AuNPs, acting as a peroxidase mimic. This material quenches the ECL signal of QDs via electrocatalytic reduction of the co-reactant H_2_O_2_. The detection principle relies on H_2_O_2_ consumption and specific recognition between concanavalin A and mannose or N-glycan residues present on the cell surface. The cytosensor exhibited a linear calibration range from 4.8 × 10^2^ to 5.0 × 10^5^ cells·mL^−1^, enabling sensitive detection of K562 cells [163].

## 7. Chemical and Structural Variability as a Limiting Factor for the Scalable Integration of Polymer–Graphene Hybrid Sensors

The scalability of fabrication strategies for polymer–graphene hybrid systems remains one of the most persistent and critical bottlenecks for the large-scale deployment of high-performance electrochemical sensors, particularly those that exploit the intrinsic multiscale heterogeneity of graphene oxide GO and reduced rGO [164]. This challenge is fundamentally rooted in the intrinsic chemical and structural variability of GO, where differences in the degree of oxidation, the spatial distribution of oxygen-containing functional groups (–CO, –COOH, –O–), and the density and nature of lattice defects give rise to a broad spectrum of materials with markedly distinct electronic structures, interfacial properties, and electrochemical behaviors [165].

These sources of variability are further exacerbated during reduction processes, as the selected reduction pathway (chemical, thermal, or electrochemical) governs not only the extent of sp^2^ network restoration but also the type and concentration of residual defects, heteroatoms, and structural disorder, ultimately defining the electrical conductivity and charge-transfer characteristics of the resulting rGO [165]. Consequently, significant dispersion is observed in key analytical performance parameters, including operating potential, sensitivity, limit of detection, response time, and tolerance to interfering species, thereby compromising reproducibility and comparability across independently fabricated sensors. An additional and increasingly relevant level of variability arises from covalent and non-covalent functionalization strategies used to integrate conductive polymers or molecular recognition elements onto GO- or rGO-based platforms [166]. In covalent approaches, reaction efficiency and surface coverage are highly sensitive to experimental conditions, including pH, the nucleophilicity of the reacting species, the stability of activated intermediates, the concentration of coupling agents, and temperature, all of which critically influence grafting density, interfacial homogeneity, and electronic coupling between components [167]. Minor deviations in these parameters can lead to substantial differences in surface chemistry, affecting both electron-transfer kinetics and analyte accessibility.

In the case of non-covalent or supramolecular assembly mechanisms (such as π–π stacking, electrostatic interactions, hydrogen bonding, or coordination with metal nanoparticles), the stability and organization of the resulting hybrid architectures are strongly dependent on ionic strength, the presence of competing species, solvent composition, and the microstructural features of the deposited films [167]. Even subtle variations in these factors can induce reorganization or partial desorption of functional components, generating chemically and electrochemically distinct interfaces [168]. Such interfacial heterogeneity not only impacts sensor sensitivity and selectivity but also affects long-term operational stability and device-to-device reproducibility, ultimately limiting the scalability and industrial translation of polymer–graphene-based electrochemical sensing technologies [169].

### From Chemical Control to Sustainability: Implications of Synthesis Routes in GO/rGO Sensors

Two sources of variability converge on these platforms: (i) the nature and chemical characteristics of the carbonaceous support (GO/rGO/doped) and (ii) the assembly kinetics of the polymer or bioreceptor on the interface [170]. In practice, small deviations in the degree of oxidation of the GO, in the defect density, or in the reduction pathway to rGO simultaneously alter the electron mobility, hydrophilicity, and anchoring capacity of functional molecules. The impact is not marginal: it manifests in the working potential, the analytical slope, and the limit of detection (LOD), which then depend on both the material chemistry and the synthesis route [171]. This sensitivity becomes especially evident when comparing architectures that rely on accessible –COOH groups (for example, EDC/NHS couplings) versus designs that prioritize π–π interactions or electrostatic assemblies. Covalent routes typically improve recognition element retention and stability under prolonged operating conditions; however, this advantage comes with a stricter dependence on the reaction microenvironment (pH, ionic strength, activation time, stoichiometric ratio, sonication intensity, and even thermal history). In both approaches, the protocol becomes a silent determinant of inter-batch variability and, by extension, of reproducibility between laboratories or in industrial production [172,173,174].

On the other hand, electropolymerization and hybrid assemblies (e.g., PEDOT or PPy on rGO with metallic nanoparticles) exhibit a marked dependence on operating parameters (applied potential, window, number of cycles, dopant type, ink viscosity, and stirring/sonication regime) that modulate effective porosity, electron percolation, and mass transport to active sites. Microvariations in these conditions result in shifted peak currents, changing overpotentials, and ultimately, calibration curves that are rarely superimposable across studies [175].

In this context, analytical performance is no longer the sole criterion for evaluating the excellence of these platforms. It is increasingly accompanied by considerations of the environmental costs of their manufacture and operation, which determine their actual viability at scale. In GO/rGO-based systems, this dimension poses a well-known dilemma between intensive oxidative routes (requiring concentrated acids and strong oxidizing agents) and alternative strategies, such as electrochemical reduction or green chemistry-inspired approaches. While the former offers relatively fine control over the density and distribution of oxygenated groups, favoring certain covalent functionalization strategies, it also generates a significant environmental burden due to the management of hazardous reagents, acidic effluents, and high energy requirements. In contrast, the latter substantially reduces environmental liabilities and simplifies scale-up by decreasing both occupational risks and treatment and control requirements. However, it often introduces greater variability in the final material properties [176,177].

This balance between chemical control and sustainability is not independent of the sources of variability discussed previously; rather, it amplifies them. The same synthesis decisions that modulate defect density, surface hydrophobicity, or molecular anchoring capacity (as reflected in shifts in working potential, analytical slope, or LOD) also determine the sensor’s environmental footprint and regulatory acceptability. From this perspective, it is pertinent that a critical review not be limited to comparing electroanalytical metrics, but rather incorporate an explicit analysis of the footprint associated with (i) the precursors and reagents used, (ii) the demand for thermal or electrical energy, (iii) the nature and volume of the effluents generated, and (iv) the occupational hazards during the synthesis and processing of the material [178].

## 8. Future Perspectives

Recent advances in electrochemical sensors demonstrate a growing use of graphene-derived nanomaterials in combination with voltammetric, amperometric, potentiometric, and electrochemiluminescence (ECL) techniques [179,180]. This convergence has enabled detection limits in the nanomolar and even femtomolar range, as demonstrated in ECL biosensors for dopamine based on Ru(bpy)_3_^2+^ nanowires integrated into rGO–Nafion films, with detection limits on the order of 10^−13^ M. However, beyond these analytical achievements, a significant gap remains between the performance reported under controlled laboratory conditions and actual functionality in clinical or environmental settings. The effective transfer of these platforms requires systematically addressing unresolved aspects, such as long-term operational stability, inter-batch reproducibility, and robustness to interference, particularly in complex matrices such as blood, food, or wastewater [181].

From a device-design perspective, there is a clear trend toward flexible, portable, and low-cost sensors, driven by developments such as graphene-based potentiometric paper electrodes that combine high mechanical flexibility with electrochemical performance comparable to conventional systems. However, the transition to these formats introduces new challenges, particularly regarding mechanical durability, active interface stability, and protection against chemical or biological contaminants. In this context, the rational incorporation of biocompatible polymer coatings and selective membranes appears as a promising strategy for extending sensor lifespan without compromising analytical sensitivity [153,182].

In addition, the integration of GO/rGO materials with microfluidics and printed electronics outlines a concrete path toward disposable platforms for rapid diagnostics and on-site monitoring. This approach not only reduces manufacturing costs and reagent consumption but also facilitates implementation in resource-constrained environments, where operational simplicity and safety are considered as important as analytical performance [183,184]. In line with the previous discussion, the future challenge lies not only in maximizing sensitivity or reducing LOD, but in designing architectures that balance performance, reproducibility, and sustainability, so that material chemistry, manufacturing protocol, and end-use context are conceived as interdependent variables within the same development strategy.

Moreover, sustainability and scalability are key factors for the next generation of sensors. Currently, many devices rely on precious metals such as Pt, Au, or Ru, which increase costs and limit large-scale production. A shift toward electrocatalysts based on abundant metals (Co, Fe, Ni) and green synthesis methods for graphene is anticipated, reducing environmental impact. Furthermore, convergence with emerging technologies—such as multimodal sensors that combine electrochemical and optical signals—will enable applications under extreme conditions and in autonomous systems for environmental monitoring, food safety, and personalized health [185,186].

## 9. Conclusions

The bibliometric analysis developed in this study, encompassing 2644 documents indexed in databases such as Scopus and Web of Science, reveals sustained growth in scientific interest in the design and fabrication of graphene-based sensors functionalized with nanomaterials. The results highlight the dominant role of countries such as China, India, and the United States, which concentrate a significant volume of scientific output and act as central nodes in international collaboration networks. The proximity and size of the nodes observed in the bibliometric maps reflect an active exchange of knowledge, infrastructure, and resources, which has driven rapid advances in materials, sensor architectures, and functionalization strategies. However, this panorama also underscores the need to strengthen synergies focused on specific problems, particularly those associated with environmental monitoring and diagnostics in contexts with economic and regulatory constraints, where technology transfer remains uneven.

From a technological standpoint, the literature review confirms that electrochemical sensors based on graphene and hybrid nanomaterials have redefined the state of the art in chemical and biomedical detection, offering devices with high sensitivity, high selectivity, and reduced response times. The integration of graphene with metallic nanoparticles, conductive polymers, and luminescent systems has significantly broadened the spectrum of applications, from quantifying biomarkers in biological fluids to monitoring environmental pollutants. However, as discussed throughout this work, analytical performance alone does not guarantee the practical viability of these platforms.

Structural challenges persist that limit its adoption outside the laboratory, including operational stability in complex arrays, inter-batch reproducibility, and susceptibility to chemical and biological interferences. These challenges are closely linked to material chemistry, GO/rGO synthesis routes, and interfacial assembly protocols, which act as silent determinants of sensor performance and reliability. In this regard, selective functionalization, rational surface design, and the incorporation of molecular recognition strategies emerge not only as tools to improve selectivity but also as key mechanisms for reducing variability and increasing device robustness.

## Figures and Tables

**Table 1:** Design of the Search Key Based on PICO Methodology Parameters.

STEP 1	Study Idea	Applications of Polymer–Graphene Composites and Functional Materials in the Design of Electrochemical Sensors				
**STEP 2**	Study problem	**P**	Polymer–graphene composites and functional materials			
		**I**	Design of polymer–graphene composites			
		**C**	Conventional methods of polymer–graphene composites and their applications			
		**O**	Development and applications of polymer–graphene composites and functional materials in the design of electrochemical sensors			
**STEP 3**	Research question	How can we design sensors that not only detect but also learn from their environment? How can we ensure reproducibility in complex biological matrices? What role do biodegradable materials play in the sustainability of these devices?				
**STEP 4**	DeCS	Polymer		DeCS		
		Graphene		Graphite *, Graphene Composites *		
		Sensors		Micro-Electrical-Mechanical System *; Systems, Micro-Electrical-Mechanical *		
		Application		Preparation *; Properties; Applications		
**STEP 5**	MeSH similarity	Polymer	Polymer, Stimuli Responsive	Responsive Polymer, Stimuli	Stimuli Responsive Polymer	Polymer, Stimuli-Responsive
		Graphene *	Graphite *	Graphene Composites		
		Sensors	Micro-Electrical-Mechanical System	System, Micro-Electrical-Mechanical	Systems, Micro-Electrical-Mechanical	Micro Electrical Mechanical Systems
		Application	Properties *	Biodegradation *	Applications *	
**STEP 6**	Search approach by variables	Polymer *	Polymer, Stimuli Responsive OR Responsive Polymer, Stimuli OR Stimuli Responsive Polymer OR Polymer, Stimuli-Responsive			
		Graphene *	Graphite * OR Graphene Composites			
		Sensors	Micro-Electrical-Mechanical System OR System, Micro-Electrical-Mechanical OR Systems, Micro-Electrical-Mechanical OR Micro Electrical Mechanical Systems			
		Application	Properties * OR Biodegradation * OR Applications *			
**STEP 7**	Advanced search key	(Polymer * OR “Polymer, Stimuli Responsive” OR “Responsive Polymer, Stimuli” OR “Stimuli Responsive Polymer” OR “Polymer, Stimuli-Responsive”) AND (Graphene * OR Graphite * OR “Graphene Composites”) AND (Sensors OR “Micro-Electrical-Mechanical System” OR “System, Micro-Electrical-Mechanical” OR “Systems, Micro-Electrical-Mechanical” OR “Micro Electrical Mechanical Systems”) AND (Application OR Properties * OR Biodegradation * OR Applications *)				

The symbol (*) indicates plural forms of the terms.

**Table 2:** Voltammetric sensors based on functionalized graphene for the detection of emerging contaminants.

Modified Electro Type	Technique	Analytes Detected	Detection Limit	Advantages/Disadvantages	Ref.
Au—GR—Cys/GCE	Square-wave voltammetry	Cd^2+^, Pb^2+^	0.10, 0.05 μg· L^−1^	Advantages: High sensitivity and low detection limits (Cd^2+^: 0.10 µg·L^−1^ and Pb^2+^: 0.05 µg·L^−1^). This is due to the synergistic effect between graphene, gold nanoparticles, and cysteine, which improves adsorption and electron transfer. Disadvantages: Its synthesis is complex, it is strongly pH-dependent, it competes for active sites, and it is costly.	[128]
GO—CO—NH—Ph/AuE	Square-wave voltammetry	Pb^2+^, Cu^2+^, Hg^2+^	3,3, 17, 17 ppb	Advantages: High sensitivity and low detection limits: Pb^2+^: 0.3 ppb (≈1 nM) Cu^2+^: 1.7 ppb (≈10 nM) Hg^2+^: 1.7 ppb (≈5 nM) Disadvantages: Significant interference from ions such as Ba^2+^, Ni^2+^, Zn^2+^, and Cd^2+^. Furthermore, it requires a critical pH (6.8) for maximum sensitivity; outside this range, the signal diminishes or becomes unstable.	[129]
rGO—Sb/GCE	Differential pulse voltammetry	Pd^2+^, Pt^2+^, Rh^3+^	0.45, 0.49, 0.49 pg· L^−1^	Advantages: High sensitivity and extremely low detection limits: Pd^2+^: 0.45 pg·L^−1^ Pt^2+^: 0.49 pg·L^−1^ Rh^3+^: 0.49 pg·L^−1^ Disadvantages: It requires multiple steps: chemical reduction, impregnation with SbNPs, stabilization with PVA, filtration, and drying, which limits scalability. Furthermore, it requires an optimal pH of 5.2.	[130]
Nafion/Hg^2+^/GO/GCE	Square-wave voltammetry	Pb^2+^	0.13 ng/L	Advantages: Wide linear range. 5–70 ng·L^−1^ 0.1–10 µg·L^−1^ Minimal interference for most ions (Cl^−^, SO_4_^2−^, NO_3_^−^, etc.). Disadvantages: Use of mercury (although immobilized, it remains a toxic material requiring careful handling) and the design is optimized for lead; performance for other metals is not reported in this study.	[131]
NH_2_—rGO/β—CD/GCE	Square-wave voltammetry	Cu^2+^	0.0028 μM	Advantages: Simple preparation and non-toxic materials: NH_2_-rGO and β-cyclodextrin are safe materials and easy to assemble on GCE. Disadvantages: Interference occurs with Pb^2+^ and Cd^2+^ at high concentrations. Furthermore, they compete for the same coordination sites, reducing the Cu^2+^ signal.	[132]
rGO/MB/Au/GCE	Differential pulse voltammetry	Fe^3+^	0.015 μM	Advantages: Synergy of materials. rGO: large surface area and active sites AuNPs: accelerate electronic transfer Methylene Blue (MB): It acts as an electronic mediator, prevents rGO aggregation, and facilitates uniform AuNP growth Disadvantages: Interference with Ag^+^ and Fe^2+^. Ag^+^ reduces the signal by AgCl formation on AuNPs Fe^2+^ increases the signal, affecting accuracy if speciation is not controlled	[133]
Au-ATPGO/GCE	Square-wave voltammetry	p-aminothiophenol	0.3 μM	Advantages: Synergy of materials. GO functionalized with p-aminophenol (ATP) provides a high density of active sites and good conductivity AuNPs improve electron transfer and sensitivity Successful determination of QR in commercial capsules and synthetic formulations without significant interferences. Disadvantages: Dependence on strict conditions. Optimal pH: 5.5 (0.1 M acetate) Instrumental parameters (frequency, amplitude, increment) must be carefully adjusted to avoid peak distortion It requires an argon purge before and during the analysis to avoid oxygen interference.	[123]

## Data Availability

No new data were created or analyzed in this study.
